# Neonatal IL-4 Over-Exposure is Accompanied by Macrophage Accumulation in Dura Mater After Instant Anti-inflammatory Cytokine Response in CSF

**DOI:** 10.1007/s10571-023-01451-4

**Published:** 2024-02-05

**Authors:** Ling Wang, Haoran Sha, Xiaoyi He, Yinyin Xie, Jiapeng Deng, Jiexuan Chen, Guoying Li, Junhua Yang

**Affiliations:** 1 Grade 2019, School of Clinical Medicine, Guangdong Pharmaceutical University, Guangzhou, Guangdong People’s Republic of China; 2 Grade 2020, School of Clinical Medicine, Guangdong Pharmaceutical University, Guangzhou, Guangdong People’s Republic of China; 3 Department of Anatomy, School of Basic Medical Sciences, Guangdong Pharmaceutical University, Higher Education Mega Center, Guangzhou, 510006 Guangdong People’s Republic of China; 4 Grade 2018, School of Clinical Medicine, Guangdong Pharmaceutical University, Guangzhou, Guangdong People’s Republic of China; 5 Guangdong Medical Association, Guangzhou, 510180 Guangdong China; 6 Guangdong Key Laboratory of Pharmaceutical Bioactive Substances, Guangdong Pharmaceutical University, Higher Education Mega Center, Guangzhou, 510006 Guangdong People’s Republic of China

**Keywords:** Cytokines, Early life, Neuroinflammation, CNS, Development, Dura mater, Macrophage

## Abstract

Multiple studies have shown that clinical events resulting into neonatal IL-4 over-exposure, such as asthma in early life and food allergy, were associated with brain damage and that the neuroinflammation induced by them might lead to cognitive impairments, anxiety-/depressive-like behaviors. IL-4 is the most major elevated cytokine in periphery when these clinical events occur and peripheral IL-4 level positively correlates with the severity of those events. Our previous studies have verified that neonatal IL-4 over-exposure induced a delayed neuroinflammatory damage in rodents, which might have adverse implications for brain development and cognition. Neuroinflammation in brain parenchyma is often accompanied by changes in CSF cytokines levels. However, whether the cytokines levels in CSF change after neonatal IL-4 over-exposure is unknown. Here, we found a delayed pro-inflammatory cytokines response (higher IL-6, IL-1β and, TNF levels) in both hippocampus and CSF after an instant anti-inflammatory cytokine response in IL-4 over-exposed rats. Moreover, the pro-inflammatory cytokines response appeared earlier in CSF than in hippocampus. The level of each of the pro-inflammatory cytokines in CSF positively correlated with that in hippocampus at the age of postnatal day 42. More microglia numbers/activation and higher M-CSF level in the hippocampus in IL-4 over-exposed rats were also observed. Furthermore, there were more macrophages with inflammatory activation in dural mater of IL-4 over-exposed rats. In sum, neonatal IL-4 over-exposure in rats induces delayed inflammation in CSF, suggesting CSF examination may serve as a potential method in predicting delayed neuroinflammation in brain following neonatal IL-4 over-exposure.

**Graphical Abstract:**

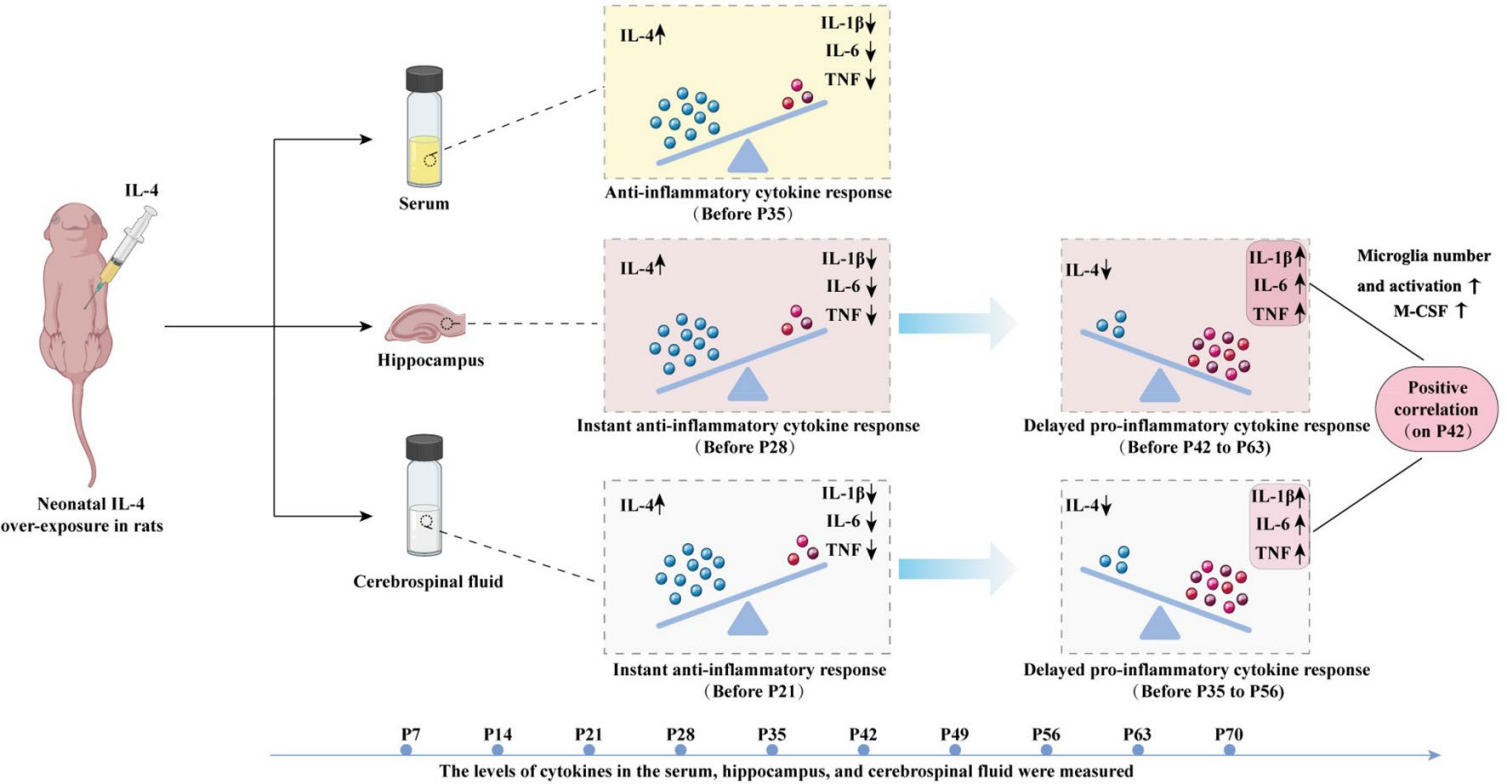

**Supplementary Information:**

The online version contains supplementary material available at 10.1007/s10571-023-01451-4.

## Introduction

In the traditional concept, interleukin (IL)-4 had been viewed as an anti-inflammatory cytokine, inducing an anti-inflammatory effect. Elevated IL-4 level means remission of neuroinflammation and neuroprotection (Wu et al. 2022b; Mali and Novotny 2022; Boccardi et al. 2018). However, the in vivo effects of IL-4 are complex. It has been recently reported that a high concentration of IL-4 has also been associated with inflammation-related diseases in newborns who showed undesirable scores on assessments of neurocognition, language, communication, and social function (Leviton et al. 2018).

There is a homeostasis between anti-inflammation and pro-inflammation in physiological condition, which plays an essential role in neural development and function. Elevated IL-4 levels due to any cause during the critical stages of brain development may break the physiological anti-inflammatory/pro-inflammatory balance in the brain, inducing strong immune activation and significantly increasing levels of pro-inflammatory cytokine which alter brain developmental trajectory and exert long-term effects on the brain (Wang et al. 2018; Bilbo et al. 2005). In addition, IL-4 may also contribute to brain damage via its ability to potentiate the effects of oxidative stress on neurons, and oxidative stress is one of the leading causes of inflammation in the central nervous systems (CNS) (Park et al. 2008). Some researchers have even supported the idea that mild-to-moderate infection and subsequent cytokine exposure during the critical stages of brain development are likely to lead to more subtle alterations, such as altering the response or vulnerability to a subsequent insult (Hagberg and Mallard 2005).

Peripheral IL-4 over-exposure is associated with neuroinflammatory damage. There are many risk factors that lead to IL-4 over-exposure in the neonatal phase and childhood, including allergic asthma, allergies to foods such as milk and eggs, bronchiolitis, and measles virus infection in the early stage (Han et al. 2016; Hammad and Lambrecht 2015; Sicherer et al. 2010; Smith et al. 2019). In addition to causing inflammation of the lungs and airways, allergic asthma can also induce strong immune activation in the brain during critical the stage of brain development, indicated by significant increase of pro-inflammatory cytokines IL-1β, IL-6, and TNF in the hippocampus and prefrontal cortex. Allergic asthma patients, therefore, are potential neuroinflammation sufferers and may develop into some nervous system diseases or mental diseases (Antunes et al. 2022; Duan et al. 2018; Han et al. 2018; Xia et al. 2014). A study by Smith et al. reported upregulated expression of the pro-inflammatory factor TNF and the activation of astrocytes in the brain of milk-allergic mice while exhibiting pronounced anxiety and depression-like behavior (Smith et al. 2019). Kordulewska NK et al. reported that children with autism showed an enhanced immune response and abnormal expression of cytokines, and the level of IL-4 was significantly increased than that in the control group, based on a comparison of blood samples from autistic patients and control participants (Kordulewska et al. 2019). An animal experimental study also shown that peripheral IL-4 penetrated the blood–brain barrier (BBB) into the CNS during neonatal period, inducing delayed hippocampal neuroinflammation and spatial cognitive impairment through down-regulation of the IL-4 receptor in the hippocampus (Wang et al. 2018).

The inflammatory status in cerebrospinal fluid (CSF) and dural mater is likely to alter similarly to the alteration in brain after neonatal peripheral IL-4 over-exposure because the immune cells and molecules in CSF and dural mater closely communicate with those in the brain parenchyma, especially when neuroinflammation occurs (Alves de Lima et al. 2020). There is no report addressing this scientific issue yet.

Addressing this scientific issue will enrich our understanding of the neurotoxic effects of neonatal IL-4 over-exposure. Moreover, this study may also provide a basic experimental clue for related clinical studies in terms of evaluation of the potential neuroinflammation in patients suffering some IL-4-inducing diseases due to the value of CSF analysis in judging the brain health status.

## Materials and Methods

### Animals and Breeding

For this study, female and male Sprague–Dawley rats (3 months of age, weighing 250–300 g) were obtained from the Guangdong Medical Laboratory Animal Center (Guangzhou, China). For mating purposes, the females were co-housed with males and were checked every day for appearance of vaginal plug or the positive vaginal smear for sperm. If any appears, then this day was considered the first day of pregnancy. The number of pregnant rats was recorded, and the pregnant rats being housed individually, with ad libitum access to food and water. All mothers and litters were housed in a specific pathogen-free condition under 12-h light/12-h dark conditions. Male neonatal rats were used in this study.

All experiments in the current study were conducted in a paired-samples experimental design, to minimize the confusing influences of non-treatment factors. To be specific, each of the pairs consisted of two male pups born to the same dam. The two pups in each pair were randomly assigned to the two different treatment conditions. Pups were sacrificed on postnatal day (P)7, P14, P21, P28, P35, P42, P49, P56, P63 or P70. The rats that were killed on P28 or later were weaned on P21. This study was approved by the Institutional Animal Ethics Committee of Guangdong Pharmaceutical University (gdpulacspf2021053) and performed in strict accordance with the U.K. Animals (Scientific Procedures) Act, 1986.

### Procedure for Neonatal Over-Exposure To IL-4

The IL-4 exposure protocol is conducted as previously described (Wang et al. 2018). Briefly, rats were injected intraperitoneally with recombinant rat IL-4(rIL-4, PeproTech, US catalog number: 400–04; 25 ng/kg in 50 μl phosphate-buffered saline (PBS) from P0 to P20; 50 ng/kg in 50 μl PBS from P22 to P34; once every other day) or a same volume of PBS.

### Collecting CSF from Anesthetized Rats

Rats were anesthetized with 2% pentobarbital, and then the animal is positioned so that the animal’s head and trunk were maintained at approximately 135◦. Prior to CSF collection, the fur on the neck of posterior to occipital of the rat was removed to reveal of skin. Next, a transverse incision is made between the ears with a sterilized ophthalmic scissors, and then a longitudinal incision perpendicular to the very center of the transverse incision was made to reveal the muscles and blunt separation of the neck-back muscles by medical forceps for the visualization of atlantooccipital fascia. The researcher used a 1-ml syringes to pierce the atlantooccipital fascia and endocranium, stopped piercing when the sense of disappearance of resistance was felt. At this time, the needle tip had entered the cisterna magna, and then slowly pulled back the syringe plunger to achieve the aspiration of CSF which was completed in less than 1 min. Acquired CSF was ejected into EP tube. The collected CSF was centrifuged at 4 °C, 6000 g for 10 min. The supernatant liquid was taken after centrifugation and then stored at—80 ℃ for subsequent analysis (Pegg et al. 2010; Nirogi et al. 2009).

### Serum Collection

After collecting CSF, the limbs of the rats were fixed quickly and then the chest was cut open by scissors to reveal the heart. Peripheral blood was collected by cardiac puncture, 0.5 ml of blood sample was collected from the right ventricle and then placed in an incubator at 37 ℃ for 1 h to coagulate. Serum was obtained by centrifugation at 4,000 g for five minutes and then stored at − 80 ℃ for subsequent analysis (Deng et al. 2021).

### Brain Tissue Homogenate Preparation

After blood collection, the rats were transventricularly perfused with 0.9% NaCl to remove blood through systemic circulation. Then hippocampus was collected immediately on ice and flushed with a PBS solution of 4 °C. The hippocampus was homogenized in RIPA lysis buffer (Beyotime, Wuhan, China; catalogue number: P0013C) containing proteinase inhibitor cocktail. After that, the homogenate was centrifuged at 13,000 g for 30 min at 4 °C, and the supernatant was isolated. A BCA protein assay kit (Beyotime, Wuhan, China; catalogue number: P0011) was used to adjust the total protein concentration of each sample to 4.5 mg/ml. The prepared samples were at—80 ℃ for subsequent analysis (Deng et al. 2021).

### Cytokines Detection

The levels of IL-4, IL-6, IL-1β, TNF, IFN-γ, IL-2, IL-8, IL-17 and M-CSF in hippocampus, CSF and serum of rats were determined using commercially available sandwich ELISA kits (Beyotime, Wuhan, China; catalogue numbers: PI615, PI303, PI328, PT516, PI510, PI577, PI548)(Immune-Biological Laboratories Co.; Fujioka, Gunma, Japan; catalogue number: 27162)(R&D Systems Inc., Minneapolis, MN, USA; catalogue number: MMC00B). According to the manufacturer’s protocol, we used the microplate reader to detect absorbance and draw standard curve, as the basis for the quantification of the measured cytokines.

### Immunofluorescence Staining and Quantification

Another batch of rats, containing CON group and IL-4 group, were set for these analyses. The animals were deeply anesthetized and then transcardially perfused with 0.9% NaCl for 5 min. After removing the mandibles and the skull rostral to the maxillae, the skull cap and the attached dura/arachnoid containing superior sagittal sinus (SSS) and two transverse sinus (TS) areas were removed with surgical scissors. Whole mounted meninges that were still attached to the skull cap were placed in 4% paraformaldehyde (PFA) in PBS and post-fixed for 24 h at 4 °C. The dura/arachnoid sample was then carefully and gently separated from the skull cap using tweezers with a thin head. The dura was then washed with PBS three times and processed for staining. After removing the skull cap, the animals were immediately transcardially perfused with ice-cold 4% PFA in PBS until muscle spasms ceased and the animal was fixed in position. The dura was blocked in PBS containing 1% bovine serum albumin and 0.25% Triton X-100 (Sigma-Aldrich, St. Louis, MO, USA; catalogue number: 93443) for 1 h at 37 °C before being incubated with primary antibodies diluted in the same blocking solution overnight at 4 °C. The primary antibodies were as follows: Chicken anti-F4/80 (Abcam, Cambridge, MA, USA; 1:200; catalogue number: ab186073) and rabbit anti-NF-κB-P65 (Bioworld Tech, MN, USA; 1:200; catalogue number BS3602). These primary antibodies used here were selected based on their brands widely reported in the literatures, which are mature and commercialized products (Secchi et al. 2017; Yu et al. 2021; Sun et al. 2021; Cheng et al. 2016). They were not self-made antibodies reagents and have undergone long-term practical usage in numerous experimental researches, with good effects reported.

Specimens were washed three times with PBS before being incubated with the following secondary antibodies for 2 h at 37 °C: FITC-conjugated donkey anti-chicken (Abcam, Cambridge, MA, USA; catalogue number: ab63507) and Alexa Fluor 555-conjugated donkey anti-rabbit (Invitrogen Molecular Probes®, Eugene, Oreg., USA; catalogue number: A-31572) antibodies. All secondary antibodies were diluted 1:400. Each of the dura samples was then placed on a glass slide with the skull face attached on the slide. This process was performed in PBS to ensure that the SSS area and two TS areas stretched without folding. Confocal micrographs of immunofluorescence staining were obtained using a LSM 780 confocal laser-scanning microscope (Zeiss, Heidelberg, Germany). Images were acquired using the Tile Scan function. All images were acquired at a resolution of 512 × 512 pixels. One image was taken per biological replicate. These images were used only for confirming that the cells of interest were successfully labeled and for observing their morphological features, not for any quantitative analysis.

The numbers of F4/80^+^, and F4/80^+^/NF-κB-P65^+^ cells were counted within the area covering both TS per animal. The total numbers of labeled cells within the selected areas were quantified using a Stereo Investigator (MicroBrightField, Williston, USA) stereology system by an investigator who was blinded to the groups. Marker placement and the reporting of total numbers of marked cells were performed using the stereology system because the dura samples were whole-mount tissues and not selected serial sections. When each of the independent areas was determined, the system provided the total numbers of all analyzed markers that represented the types of counted cells.

For estimating the Iba-1^+^ cells in the hippocampus, free-floating sections were washed in 0.1 m PBS and then incubated in PBS containing 1% bovine serum albumin, and 0.25% Triton X-100 (Sigma-Aldrich) for 1 h. The specimens were then incubated overnight at 4 ◦C with rabbit anti-Iba-1 (1:1000; Wako Chemicals). The sections were washed three times before incubated at 37 ℃ for 2 h with secondary antibodies, Alexa Fluor 488-conjugated goat anti-rabbit (Invitrogen, 1:400). The labeled cells were quantified as previously described (Wang et al. 2018). The unilateral hippocampus of each animal were estimated using a stereology system, Stereo Investigator (MicroBrightField, Williston, USA). For each hippocampus, a total of ten coronal sections equidistantly spanning the entire hippocampus were used. After measuring the actual section thickness, the appropriate guard zones both at the top and the bottom of each of the sections were defined to avoid oversampling. Counting frames of 15 × 15 × 20 μm were set in a 40 × 40 μm matrix that were randomly superimposed onto the regions of interest. The coefficient of error in all estimation were less than 0.10. Zeiss LSM 780 confocal laser-scanning microscope was used to capture the representative confocal micrographs of labeled cells.

### Statistical Analyses

Data analyses were performed by using SPSS 26.0 software (IBM Company, Armonk, NY, USA) and Graph Prism software. Data were firstly tested for normality and variance heterogeneity by Shapiro–Wilk test and Levene test, respectively.

**Fig. 1:**
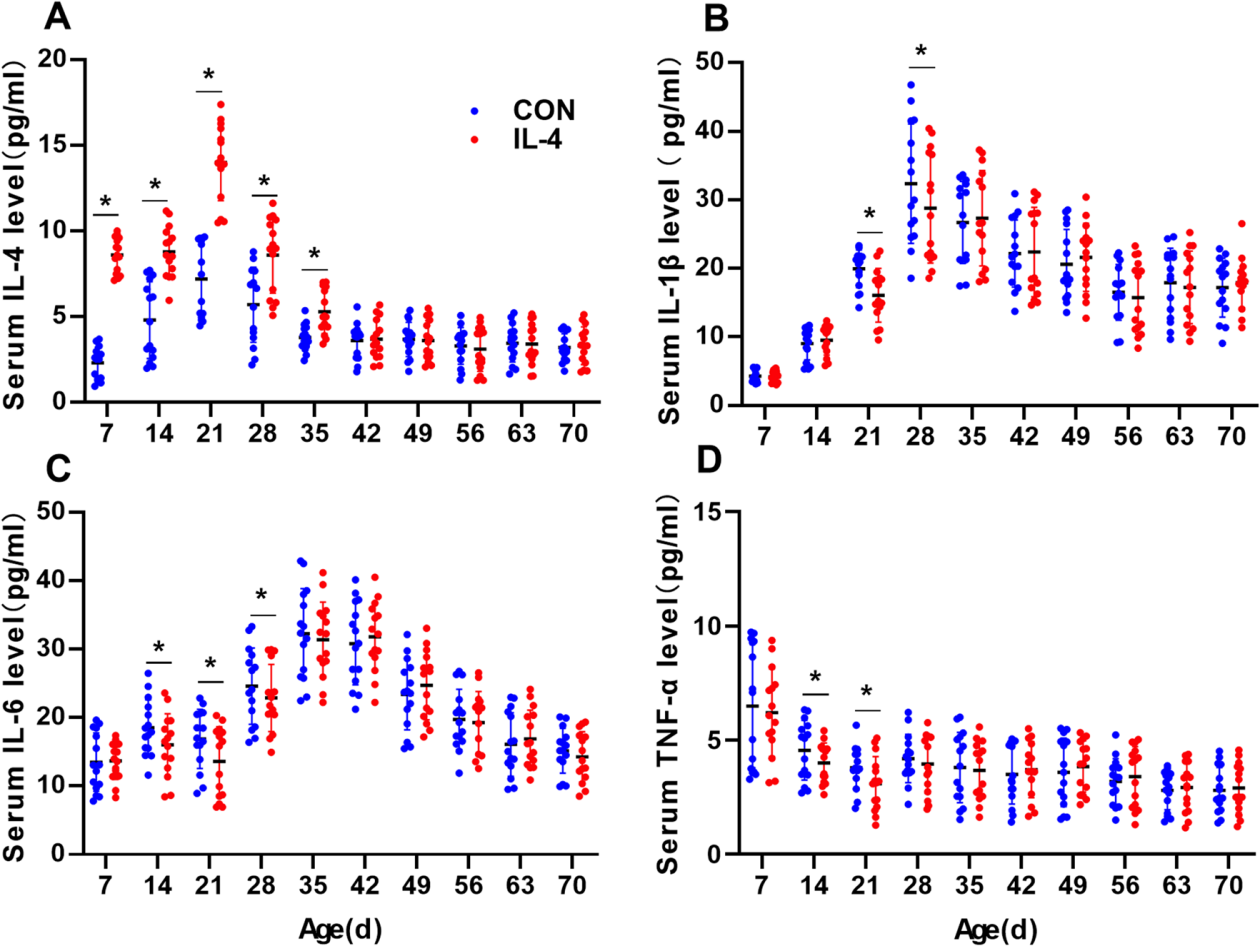
Neonatal IL-4 over-exposure elevated the IL-4 level and reduced the pro-inflammatory cytokine levels in the serum before P35. (**A**–**D**) The dots represent the average levels of IL-4 (**A**), IL-1β (**B**), IL-6 (**C**) and TNF (**D**) in the serum. The data represent the means ± SD. *p value < Bonferroni–Holm α value; *n* = 15/group; Paired samples *t*-test was used

**Fig. 2:**
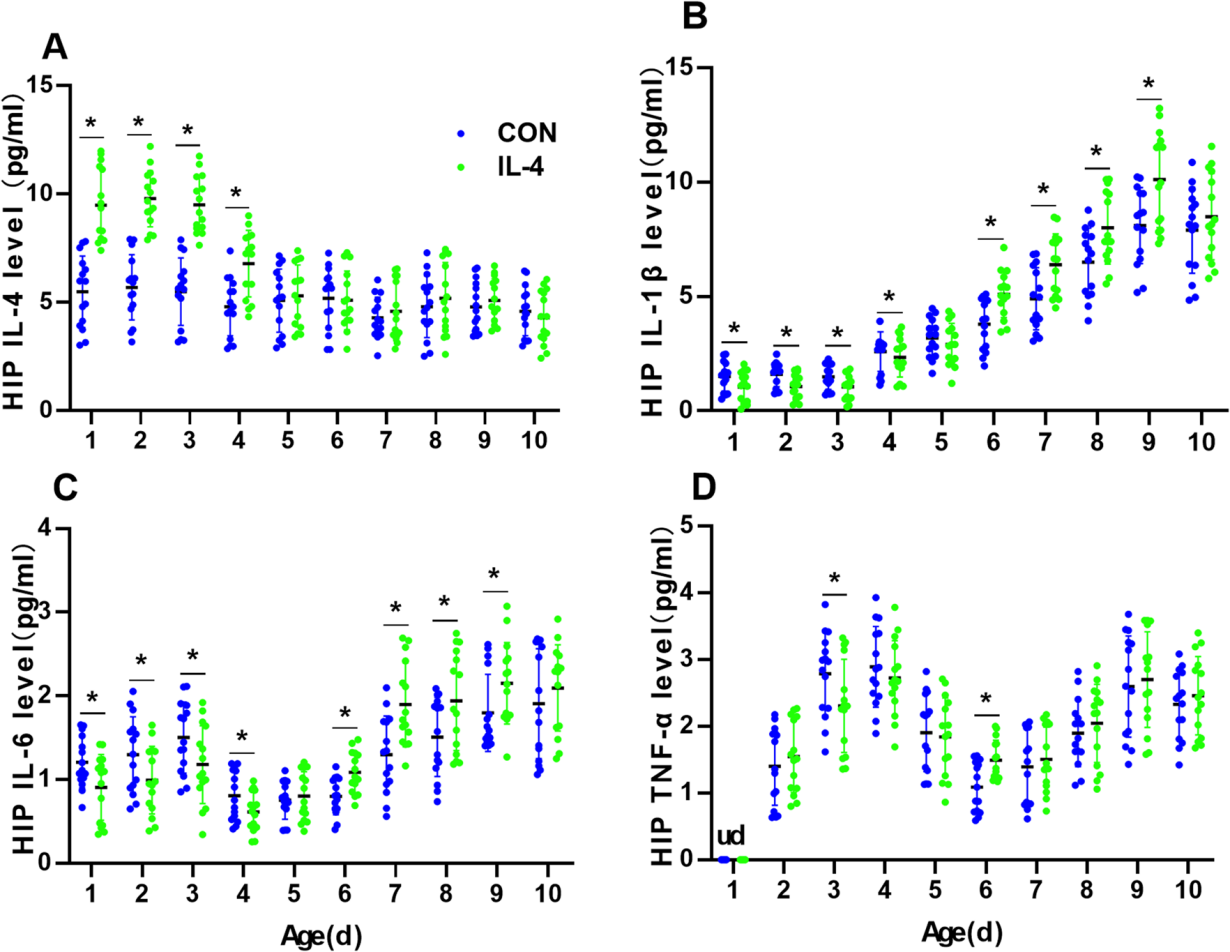
Neonatal IL-4 over-exposure induced an instant anti-inflammatory effect and a subsequent pro-inflammatory effect in the hippocampus. (**A**–**D**) The dots represent the average levels of IL-4 (**A**), IL-1β (**B**), IL-6 (**C**) and TNF (**D**) in the hippocampus. The data represent the means ± SD. *p value < Bonferroni–Holm α value; *n* = 15/group; Paired samples *t*-test was used. *HIP* hippocampus; *ud* undetectable

**Fig. 3:**
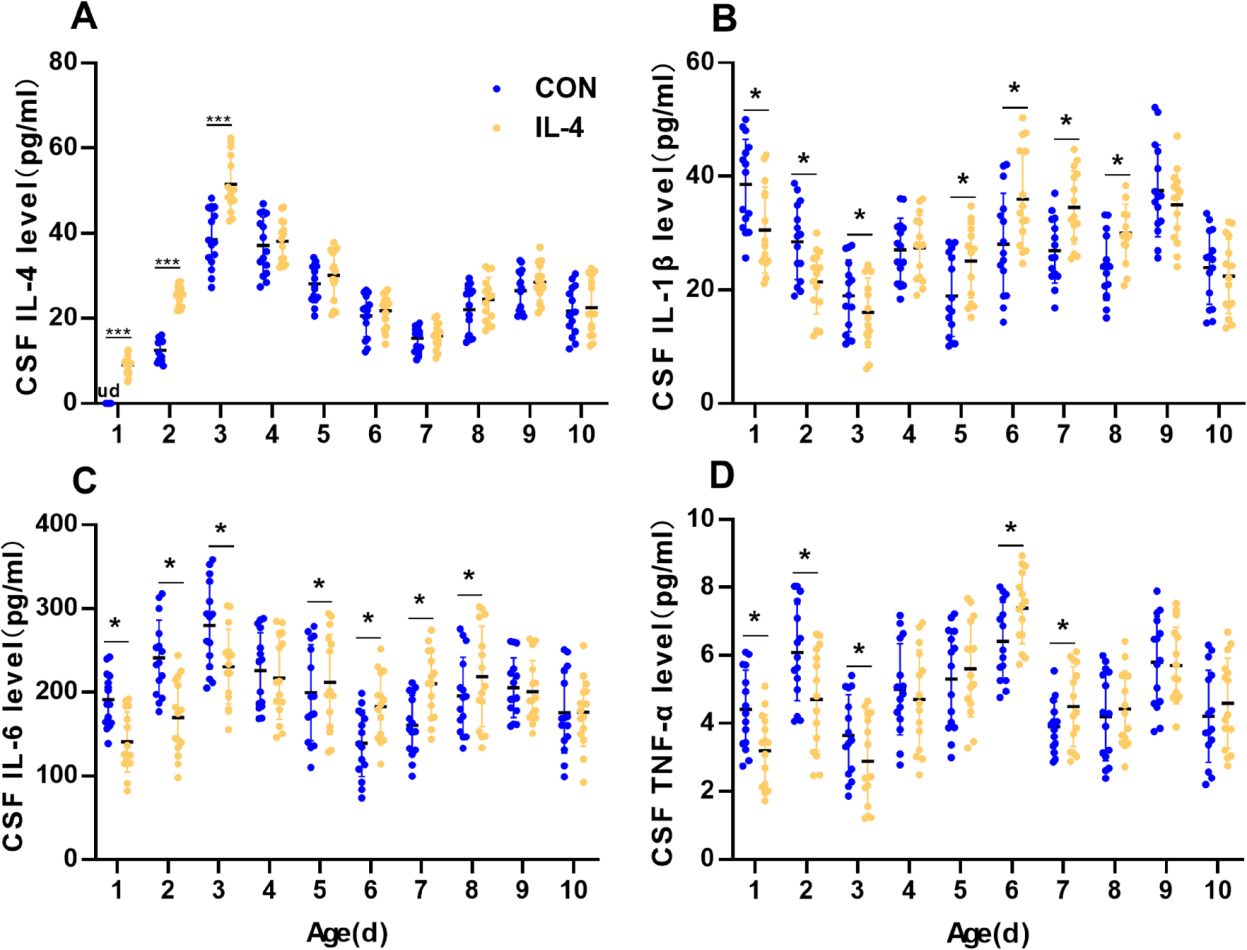
Neonatal IL-4 over-exposure induced an instant anti-inflammatory effect and a subsequent pro-inflammatory effect in the CSF. (**A**–**D**) The dots represent the average levels of IL-4 (**A**), IL-1β (**B**), IL-6 (**C**) and TNF (**D**) in the cerebrospinal fluid. The data represent the means ± SD. *p value < Bonferroni–Holm α value; *n* = 15/group; Paired samples *t*-test was used. *CSF* cerebrospinal fluid; *ud* undetectable

The Paired-samples test, also known as the dependent sample test is one of the widely used statistical tools to determine statistical difference between two measurements or two conditions (Gaddis and Gaddis 1990). All experiments in the current study were conducted in a paired samples experimental design, to minimize the confusing influences of non-treatment factors, such as gene background, sex, rearing behaviors. Therefore, the data in the current study were statistically analyzed using paired test to determine the effects by treatment factor (neonatal IL-4 over-exposure). According to the statistical principles, the normality of the differences calculated within the two measured values from the two samples in each of the pairs were first analyzed using the Shapiro–Wilk test to: if normality is accepted, the paired t-test (parameter test) is used; otherwise the Wilcoxon signed ranked test (non-parametric test) is used. Since multiple statistical analyses on the same outcome parameter, e.g., IL-4 serum levels, with different groups (each time point is a new group) were performed, we adjusted for multiple comparisons to avoid that the type I error would increase with each statistical test performed using Bonferroni-Holm correction in Fig. 1–3 and Fig. 8.

**Fig. 4:**
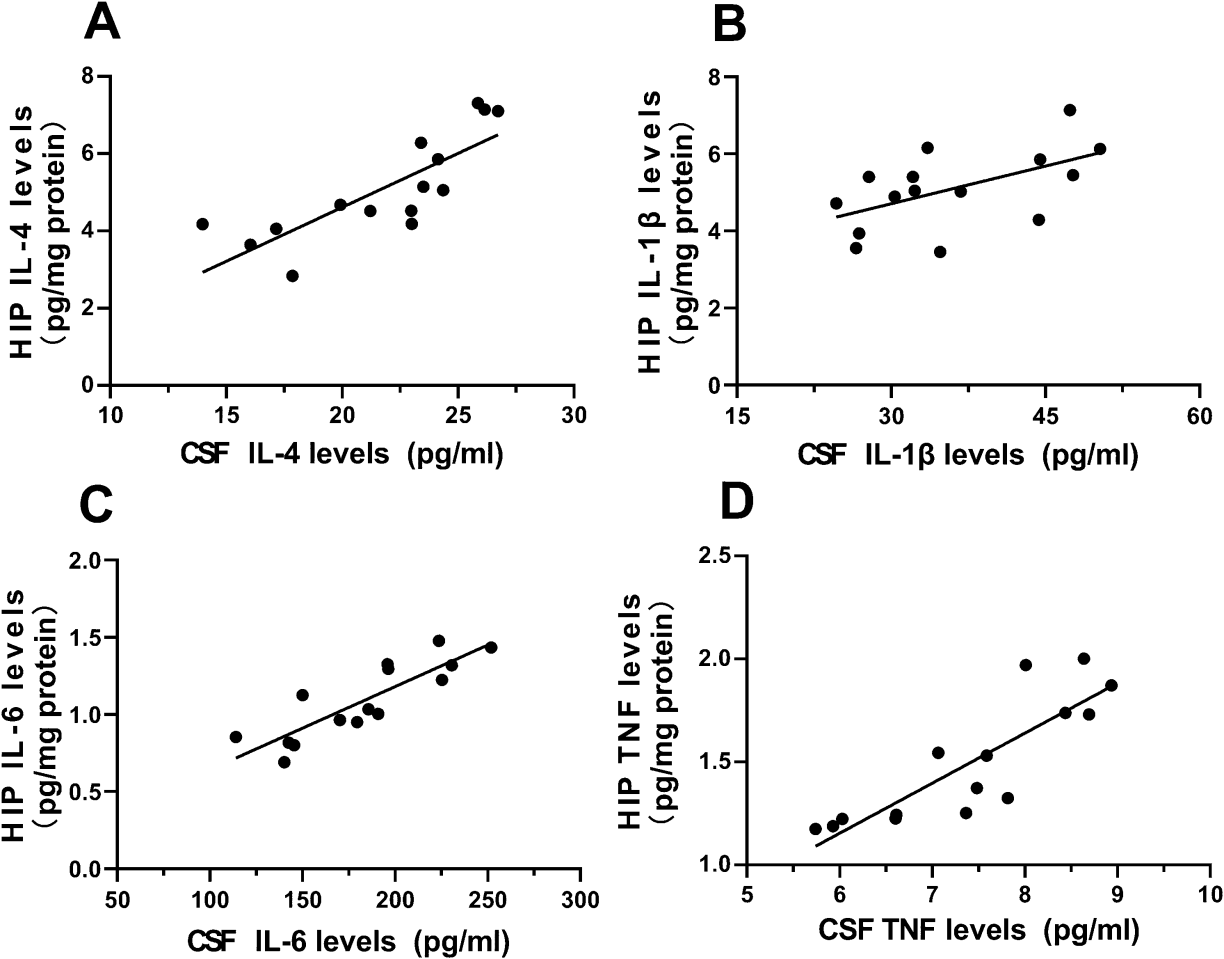
The positive correlation of the levels of IL-4, IL-1β, IL-6 and TNF in CSF with the levels of IL-4, IL-1β, IL-6 and TNF in hippocampus in IL-4 group. **A** Correlation analyses between the CSF IL-4 level and the hippocampal levels of IL-4. **B** Correlation analyses between the CSF IL-1β level and the hippocampal levels of IL-1β. **C** Correlation analyses between the CSF IL-6 level and the hippocampal levels of IL-6. **D** Correlation analyses between the CSF TNF level and the hippocampal levels of TNF. *n* = 15/group; Pearson’s correlation analysis. *CSF* cerebrospinal fluid; *HIP* hippocampus

**Fig. 5:**
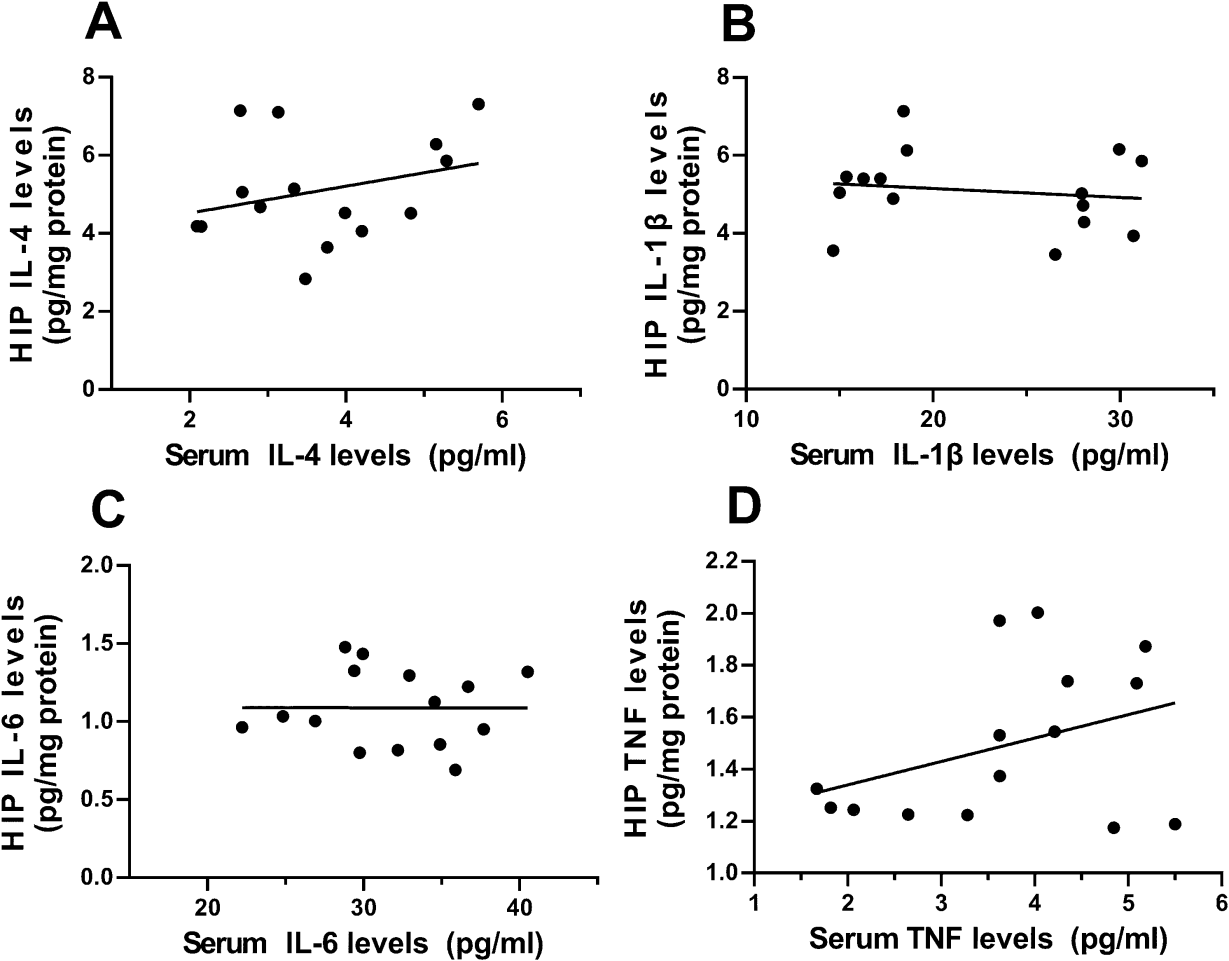
The positive correlation of the levels of IL-4 in serum with those in hippocampus in IL-4 group. **A** Correlation analyses between the serum IL-4 level and the hippocampal levels of IL-4. **B** Correlation analyses between the serum IL-1β level and the hippocampal levels of IL-1β. **C** Correlation analyses between the serum IL-6 level and the hippocampal levels of IL-6. **D** Correlation analyses between the serum TNF level and the hippocampal levels of TNF. *n* = 15/group; Pearson’s correlation analysis. *HIP* hippocampus

**Fig. 6:**
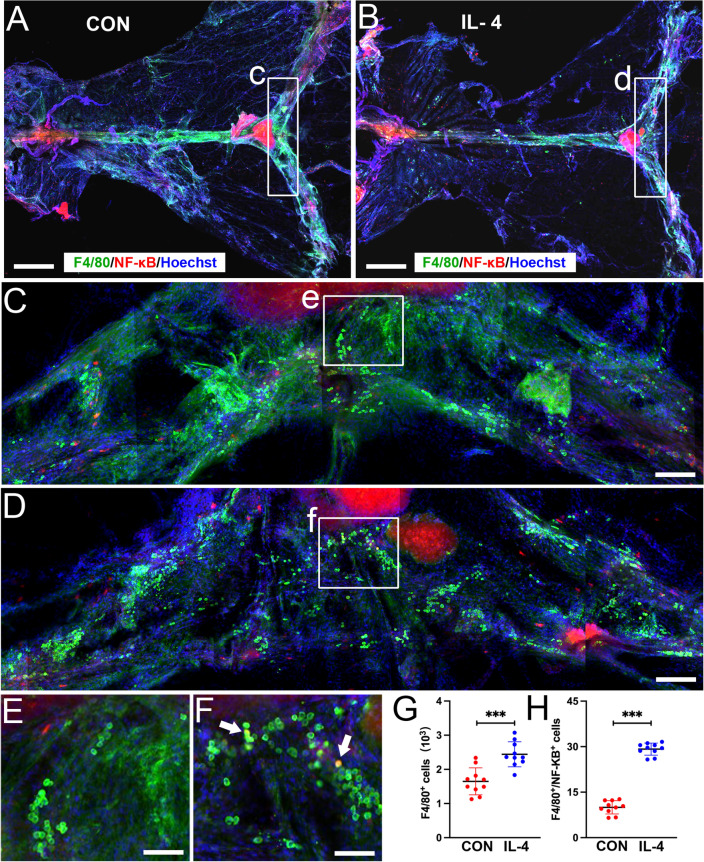
More F4/80^+^ and F4/80^+^/NF-κB^+^ cells in dural mater of IL-4 group. **A**, **B** Images showing the whole dural mater and the selected scopes shown as C and D in higher magnification. **C**, **D** Images showing the selected scopes containing the medial parts of both transverse sinuses and the selected scopes shown as E and F in higher magnification. **E**, **F** Images showing the selected scopes containing the parts connecting both transverse sinuses where exist some F4/80^+^/NF-κB^+^ co-labeled cells (green: F4/80^+^; red: NF-κB; blue: nuclei). Scale bars, 500 μm in (**A**, **B**), 100 μm in (**C**, **D**), 50 μm in (**E**, **F**). (**G**, **H**) Dots represent the numbers of F4/80^+^ and F4/80^+^/NF-κB^+^ cells in each group of rats. *n* = 10 rats/group. ***: *p* < 0.001. Paired samples *t*-test. Data are presented as the mean ± SD

**Fig.7:**
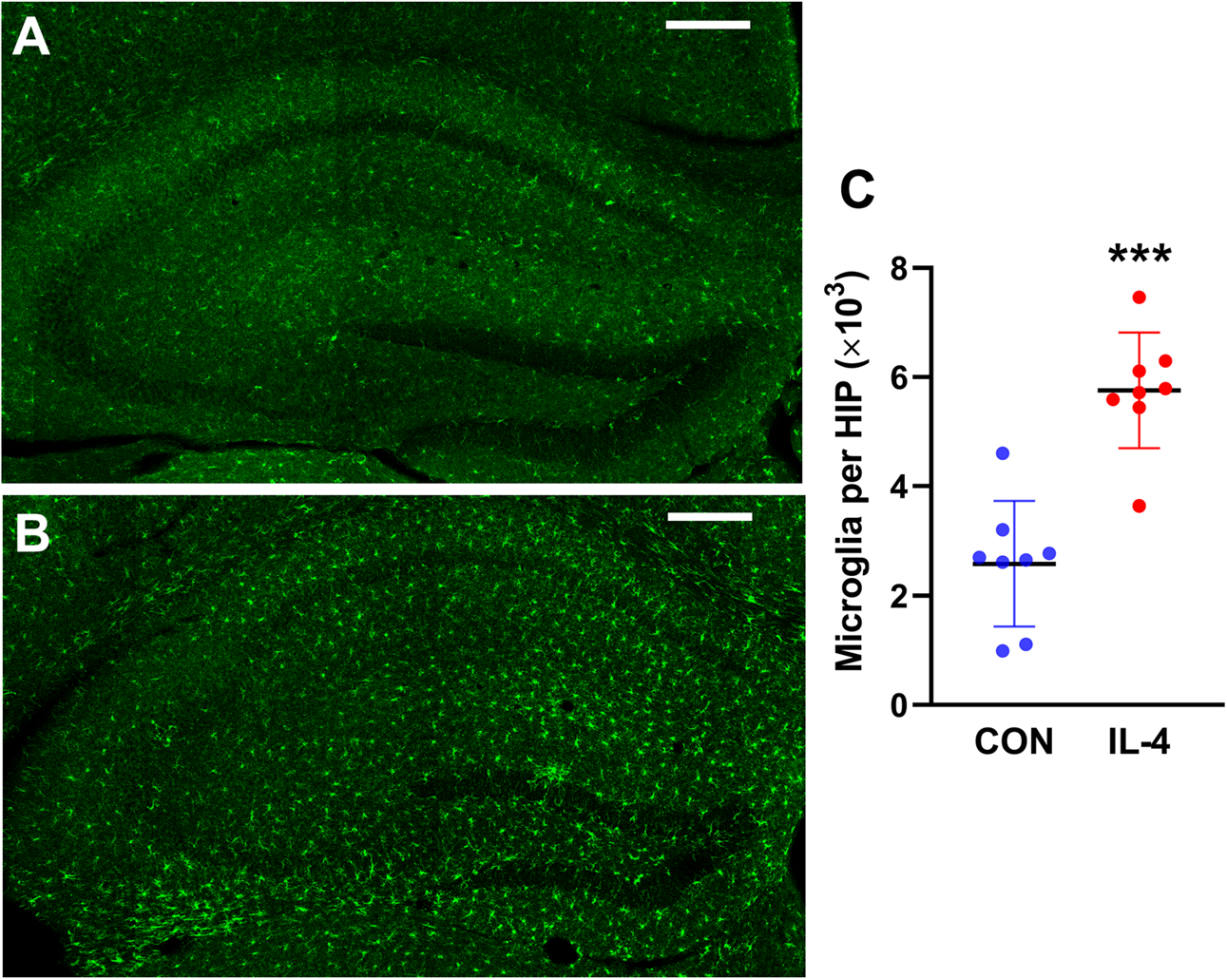
Neonatal IL-4 over-exposure increased significantly the number of microglia in the hippocampus and induced their activation on P49. **A**, **B** Representative micrographs showing the microglia (green signal: Iba-1^+^) in the hippocampus in CON rats (**A**) and IL-4 rats (**B**) at the age of P49. Scale bar, 100 μm. **C** Data represent the numbers of microglia in the hippocampus of each group of rats. The data in (**C**) represent the means ± SD. *** *p* < 0.001; *n* = 8 rats/group; Paired samples *t*-test. *HIP* hippocampus

**Fig. 8:**
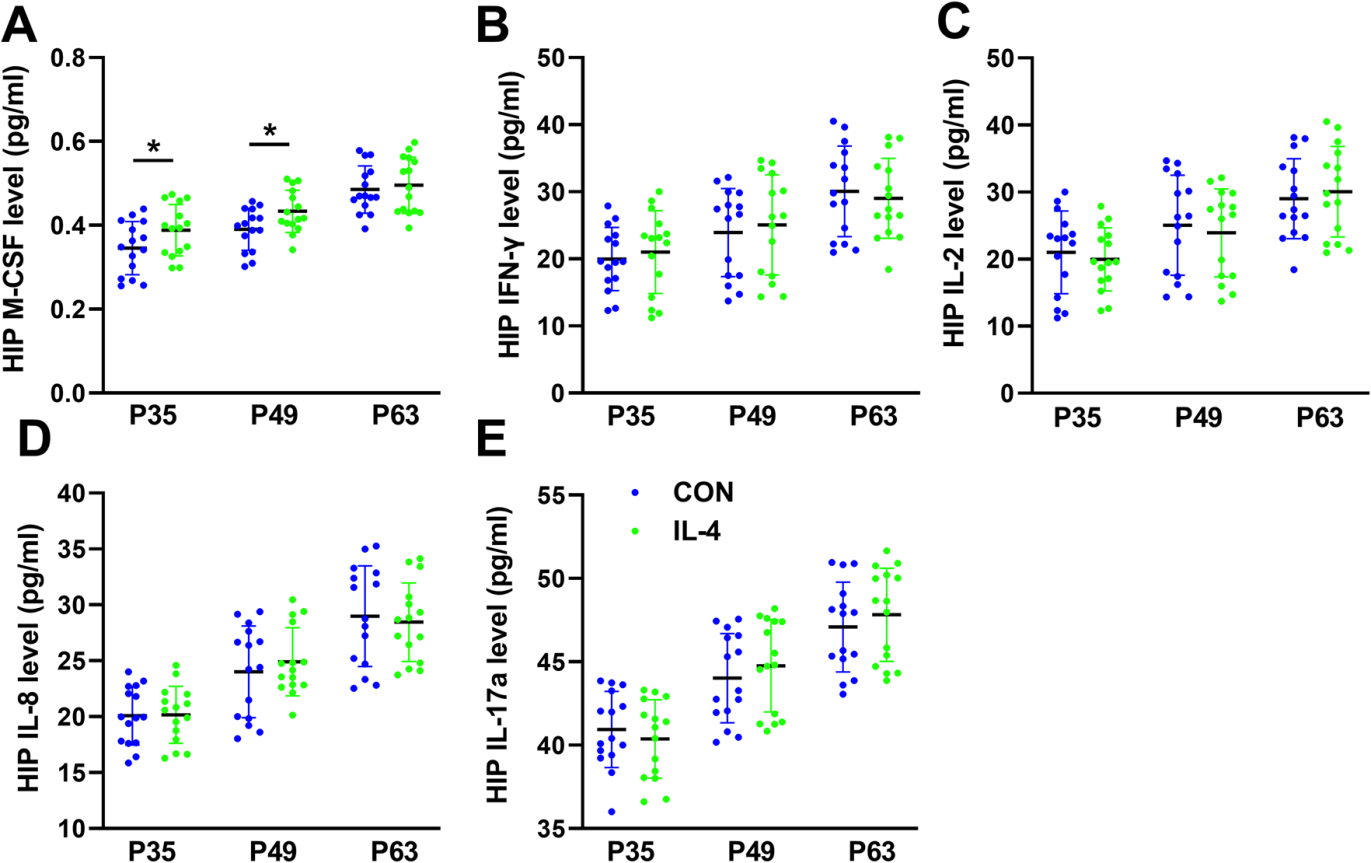
Neonatal IL-4 over-exposure increased M-CSF slightly in the hippocampus on P35 and P49. (**A**–**E**) The dots represent the average levels of each of the cytokines in the hippocampus. The data represent the means ± SD. *p value < Bonferroni–Holm α value; *n* = 15/group; Paired samples *t*-test was used. *HIP* hippocampus

Although some data were analyzed using a non-parametric test due to the unmoral distribution of the differences calculated within the two measured values from the two samples in each of the pairs, the distributions of the measured values from the samples in each group were still normal. According to the statistical principles, these groups of data are expressed in the figures (Fig. 1–3, Fig. 6–8) as means ± SD (standard deviation). The data in Fig. 4–5 were analyzed by using Pearson’s correlation. *p* < 0.05 was considered to indicate a statistically significant difference. All statistical details of the results of all statistical tests, including tests for normality and comparison, were reported in the Supplementary Material. This study was lack of a priori sample size calculation. The sample size of the experimental design was determined based on our experience (Wang et al. 2018) and reasonable value of SD. And the sample sizes in our experiments were similar to those generally used in the experimental studies in the same field (Blondel et al. 2022).

## Results

### Neonatal IL-4 Over-Exposure Elevated IL-4 Levels and Reduced the Pro-Inflammatory Cytokine Levels in Serum before P35

Firstly, we observed the effect of IL-4 over-exposure in neonatal rats on the levels of IL-4 and three classic pro-inflammatory cytokines in serum from the neonatal period to adulthood (P70). Specifically, the levels of IL-4, IL-1β, IL-6 and TNF on P7, P14, P21, P28, P35, P42, P49, P56, P63 and P70 in the serum of IL-4 group and CON group were measured.

Data in Fig. 1 present the levels of cytokines in the serum from the neonatal period to adulthood. The levels of IL-4 in serum of IL-4 group were significantly higher than CON group from P7 to P35, with no significant differences between the two groups from P42 to P70 (Paired samples *t*-test with Bonferroni-Holm correction; P7, *p* < 0.001, Bonferroni–Holm *α* = 0.005; P14, *p* < 0.001, Bonferroni–Holm *α* = 0.0071; P21, *p* < 0.001, Bonferroni–Holm *α* = 0.0056; P28, *p* < 0.001, Bonferroni–Holm *α* = 0.0063; P35, *p* < 0.001, Bonferroni–Holm *α* = 0.0083; *n* = 15; all *p* values > Bonferroni–Holm α values; *n* = 15; Fig. 1A).

The levels of IL-1β in serum of IL-4 group showed a downward trend before P28 compared to CON group with statistically significant differences on P21 and P28, with no significant differences at the other ages (Paired samples *t*-test with Bonferroni-Holm correction; P21, *p* = 0.005, Bonferroni–Holm *α* = 0.0056; P28, *p* = 0.004, Bonferroni–Holm *α* = 0.0050; all *p* values for the other time points > Bonferroni–Holm α values; *n* = 15; Fig. 1B).

The levels of IL-6 in serum of IL-4 group showed a downward trend before P28 compared to CON group with statistically significant differences on P14, P21 and P28, with no significant differences at the other ages (Paired samples *t*-test with Bonferroni-Holm correction; P14, *p* = 0.002, Bonferroni–Holm *α* = 0.0063; P21, *p* < 0.001, Bonferroni–Holm *α* = 0.0050; P28, *p* = 0.002, Bonferroni–Holm *α* = 0.0056; all *p* values for the other time points > Bonferroni–Holm α values; *n* = 15; Fig. 1C).

The levels of TNF showed mild decreases than CON group on P14 and P21, with no significant alterations at other ages (Paired samples *t*-test with Bonferroni-Holm correction; P14, *p* = 0.004, Bonferroni–Holm *α* = 0.0056; P21, *p* = 0.002, Bonferroni–Holm *α* = 0.0050; all *p* values for the other time points > Bonferroni–Holm α values; *n* = 15; Fig. 1D).

To summarize, the results shown above indicate that the decreased levels of pro-inflammatory cytokines is accompanied by elevation of the peripheral IL-4 levels.

### Neonatal IL-4 Over-Exposure Induced an Instant Anti-Inflammatory Effect and a Subsequent Pro-Inflammatory Effect in the Hippocampus

Having observed the effect of neonatal IL-4 over-exposure on the levels of IL-4, IL-1β, IL-6, and TNF in the serum, we next measured the levels of these cytokines in the hippocampus at every same age point.

Data in Fig. 2 present the levels of cytokines in the hippocampus from the neonatal period to adulthood. The IL-4 levels in hippocampus of IL-4 group were significantly higher than CON group from P7 to P28(Paired samples *t*-test with Bonferroni-Holm correction; P7, *p* < 0.001, Bonferroni–Holm *α* = 0.063; P14, *p* < 0.001, Bonferroni–Holm *α* = 0.0056; P21, *p* < 0.001, Bonferroni–Holm *α* = 0.0050; P28, *p* < 0.001, Bonferroni–Holm *α* = 0.0071; *n* = 15; Fig. 2A), with no significant differences between the two groups from P35 to P70 (Paired samples *t*-test with Bonferroni-Holm correction, all *p* values > Bonferroni–Holm α values; *n* = 15; Fig. 2A).

Both IL-6 and IL-1β levels showed a downward trend in IL-4 group from P7 to P28, with statistically significant decreases compared to CON group but showed a period of rebounding increases from P42 to P63 compared to CON group, with no significant differences between the two groups on P35 and P70 (IL-1β: Paired samples *t*-test with Bonferroni-Holm correction; P7, *p* < 0.001, Bonferroni–Holm *α* = 0.0125; P14, *p* < 0.001, Bonferroni–Holm *α* = 0.0071; P21, *p* < 0.001, Bonferroni–Holm *α* = 0.0083; P28, *p* < 0.001, Bonferroni–Holm *α* = 0.0167; P35, *p* = 0.071, Bonferroni–Holm *α* = 0.0250; P42, *p* < 0.001, Bonferroni–Holm *α* = 0.0063; P49, *p* < 0.001, Bonferroni–Holm *α* = 0.0050; P56, *p* < 0.001, Bonferroni–Holm *α* = 0.0100; P63, *p* < 0.001, Bonferroni–Holm *α* = 0.0056; P70, *p* = 0.390, Bonferroni–Holm *α* = 0.0500; *n* = 15; Fig. 2B) (IL-6: Paired samples *t*-test with Bonferroni-Holm correction; P7, *p* < 0.001, Bonferroni–Holm *α* = 0.0083; P14, *p* < 0.001, Bonferroni–Holm *α* = 0.0071; P21, *p* < 0.001, Bonferroni–Holm *α* = 0.0067; P28, *p* < 0.001, Bonferroni–Holm *α* = 0.0100; P35, *p* = 0.278, Bonferroni–Holm *α* = 0.500; P42, *p* < 0.001, Bonferroni–Holm *α* = 0.0050; P49, *p* < 0.001, Bonferroni–Holm *α* = 0.0056; P56, *p* < 0.001, Bonferroni–Holm *α* = 0.0063; P63, *p* < 0.001, Bonferroni–Holm *α* = 0.0125; P70, *p* = 0.087, Bonferroni–Holm *α* = 0.0250; *n* = 15; Fig. 2C). From P14 to P70, the TNF level showed a tendency in similar to the IL-6 and IL-1β levels in IL-4 group, but only two statistically significant differences were found on P21 and P42, with no significant alterations at other ages (Paired samples *t*-test with Bonferroni-Holm correction; P21,* p* < 0.001, Bonferroni–Holm *α* = 0.0056; P42, *p* = 0.004, Bonferroni–Holm *α* = 0.0063; all *p* values for the other time points > Bonferroni–Holm α values; *n* = 15; Fig. 2D).

All of these suggested that neonatal IL-4 over-exposure induced an instant anti-inflammatory effect in the preceding 28 days and a subsequent pro-inflammatory effect that lasted from P42 to P63 in the hippocampus of IL-4 group, which consists with the previous study (Wang et al. 2018).

### Neonatal IL-4 Over-Exposure Induced an Instant Anti-Inflammatory Effect and a Subsequent Pro-Inflammatory Effect in the CSF, Which is Phenomenally Similar to Hippocampus, but the Pro-Inflammatory Cytokine Response Appeared Relatively Earlier

Neuroinflammation is constantly accompanied by changes in CSF cytokines. Therefore, we hypothesized that changes in CSF cytokines could reflect the progress of neuroinflammation earlier. To verify this hypothesis, we then measured levels of IL-4, IL-1β, IL-6 and TNF in the CSF of neonatal IL-4 over-exposure rats to investigate the characteristics of CSF cytokine changes and the relation between cytokine changes of CSF and hippocampus.

Data in Fig. 3 present the levels of cytokines in the CSF from the neonatal period to adulthood. The IL-4 levels were significantly higher in the CSF of IL-4 group compared to that of CON group from P7 to P21 (Paired samples *t*-test with Bonferroni-Holm correction. P7, *p* < 0.001, Bonferroni–Holm *α* = 0.063; P14, *p* < 0.001, Bonferroni–Holm *α* = 0.0050; P21, *p* < 0.001, Bonferroni–Holm *α* = 0.0056; all the other *p* values > Bonferroni–Holm α values; *n* = 15; Fig. 3A), with no significant differences from P28 to P70 (Paired samples *t*-test with Bonferroni-Holm correction. all the *p* values > Bonferroni–Holm α values; *n* = 15; Fig. 3A).

Just like in the hippocampus, the levels of IL-1β, IL-6 and TNF in the CSF showed a anti-inflammatory profile in IL-4 group from P7 to P21 and a pro-inflammatory profile from P35, with a eventually returning to the normal levels at P70. (IL-1β: Paired samples *t*-test with Bonferroni-Holm correction; P7, *p* < 0.001, Bonferroni–Holm *α* = 0.0056; P14, *p* < 0.001, Bonferroni–Holm *α* = 0.0050; P21, *p* < 0.001, Bonferroni–Holm *α* = 0.0125; P28, *p* = 0.0572, Bonferroni–Holm *α* = 0.0050; P35, *p* = 0.071, Bonferroni–Holm *α* = 0.0083; P42, *p* < 0.001, Bonferroni–Holm *α* = 0.0071; P49, *p* < 0.001, Bonferroni–Holm *α* = 0.0063; P56, *p* < 0.001, Bonferroni–Holm *α* = 0.0100; P63, *p* = 0.0300, Bonferroni–Holm *α* = 0.0167; P70, *p* = 0.476, Bonferroni–Holm *α* = 0.0250; *n* = 15; Fig. 3B).

(IL-6: Paired samples *t*-test with Bonferroni-Holm correction; P7, *p* < 0.001, Bonferroni–Holm *α* = 0.0063; P14, *p* < 0.001, Bonferroni–Holm *α* = 0.0050; P21, *p* < 0.001, Bonferroni–Holm *α* = 0.0083; P28, *p* = 0.0700, Bonferroni–Holm *α* = 0.0167; P35, *p* = 0.071, Bonferroni–Holm *α* = 0.0125; P42, *p* < 0.001, Bonferroni–Holm *α* = 0.0071; P49, *p* < 0.001, Bonferroni–Holm *α* = 0.0056; P56, *p* < 0.001, Bonferroni–Holm *α* = 0.0100; P63, *p* = 0.3680, Bonferroni–Holm *α* = 0.0250; P70, *p* = 0.8760, Bonferroni–Holm *α* = 0.0500; *n* = 15; Fig. 3C).

(TNF: Paired samples *t*-test with Bonferroni-Holm correction; P7, *p* < 0.001, Bonferroni–Holm *α* = 0.0050; P14, *p* < 0.001, Bonferroni–Holm *α* = 0.0056; P21, *p* < 0.001, Bonferroni–Holm *α* = 0.0071; P28, *p* = 0.2005, Bonferroni–Holm *α* = 0.0167; P35, *p* = 0.1486, Bonferroni–Holm *α* = 0.0100; P42, *p* < 0.001, Bonferroni–Holm *α* = 0.0063; P49, *p* = 0.0083, Bonferroni–Holm *α* = 0.0083; P56, *p* = 0.1663, Bonferroni–Holm *α* = 0.0125; P63, *p* = 0.5381, Bonferroni–Holm *α* = 0.0500; P70, *p* = 0.5330, Bonferroni–Holm *α* = 0.0250; *n* = 15; Fig. 3D).

These results hinted that the neonatal IL-4 over-exposure induced an anti-inflammatory effect in the preceding 21 days and a subsequent pro-inflammatory effect that lasted from P35 to P56 in the CSF in IL-4 group. Moreover, the pro-inflammatory cytokine response in CSF appeared relatively earlier than in the hippocampus.

### The Levels of Pro-Inflammatory Cytokines in Hippocampus Positively Correlated to Those in CSF at the Individual Level on P42

All the above results suggest that delayed neuroinflammation occurred both in the CSF and hippocampus of IL-4 group at all or some of the age points after P35 or P42, indicated by IL-1β, IL-6 and TNF altered significantly at the two sites. To further explore the relationship of the alterations of four cytokines in the CSF to that in the hippocampus, correlation analyses at the individual level were then performed between the hippocampus level and the CSF level of each of the four cytokines we measured on P42. Both CSF and hippocampus are in the pro-inflammatory cytokine response on P42.

IL-4 could spread to the brain through the choroid plexus, promoting the production of proinflammatory cytokines by microglia and affects inflammation status there.Therefore, we also correlated the cytokine levels in the hippocampus with those in blood at P42.

The results showed that levels of the three pro-inflammatory cytokines in CSF were positively correlated with those in hippocampus on P42 (Pearson’s correlation coefficient; IL-1β: *r*^*2*^ = 0.3073, *p* < 0.0320; IL-6: *r*^*2*^ = 0.7360, *p* < 0.001; TNF: *r*^*2*^ = 0.7245, *p* < 0.001; *n* = 15; Fig. 4B–D). The levels of IL-4 in CSF and hippocampus also have a positive correlation (Pearson’s correlation coefficient; IL-4: *r*^*2*^ = 0.6473, *p* < 0.01; *n* = 15; Fig. 4A).

As for the correlation analyses between the cytokine levels in serum with those in hippocampus, our results showed that there was no significant correlation for any cytokines between serum and hippocampal levels (Pearson’s correlation coefficient; IL-4: *r*^*2*^ = 0.0844, *p* = 0.2934; IL-1β: *r*^*2*^ = 0.0216, *p* = 0.6010; IL-6: *r*^*2*^ < 0.0001, *p* = 0.9934; TNF: *r*^*2*^ = 0.1368, *p* = 0.1748; *n* = 15; Fig. 5).

### Neonatal IL-4 Over-Exposure Induced Macrophage Accumulation in Dura Mater after Instant Anti-Inflammatory Cytokine Response in CSF

It has been reported that macrophages in dura mater are an important source of cytokines in cerebrospinal fluid, and their number and activation state are changed by the regulation of cytokines in cerebrospinal fluid (Wieseler-Frank et al. 2007; Reuter et al. 2001). Therefore, macrophages in dura mater were observed at P42 when pro-inflammatory effect appeared in the CSF and hippocampus in IL-4 group. NF-κB signaling is the major pathway by which macrophage polarize towards M1 phenotype and F4/80^+^/NF-κB^+^ cells represent pro-inflammatory activation profile of macrophages (Wu et al. 2022a; Wang et al. 2014; Zhou et al. 2023). So we also observed the expression of NF-κB in dural macrophages by double labeling using immunofluorescence staining.

There were more F4/80^+^ and F4/80^+^/NF-κB^+^ cells in dural mater of IL-4 over-exposed rats (Paired samples *t*-test; *p* < 0.001; *n* = 10; Fig. 6) than in CON group (Paired samples *t*-test; *p* > 0.05; *n* = 10; Fig. 6). These data indicated more total macrophages as well as more pro-inflammatory activated macrophages in dura mater IL-4 over-exposed rats at P42.

### Neonatal IL-4 Over-Exposure Increased Significantly the Number of Microglia in the Hippocampus and Induced their Activation on P49

The numbers and activation of microglia in the hippocampus have also been observed on P49 when the pro-inflammatory cytokines had a largest extent of increase as indicated by the data shown in Fig. 2. As shown in Fig. 7, there were significantly more microglia (Paired samples *t*-test., *t* = 6.25; *p* < 0.001; *n* = 8 rats/group) in the hippocampus of the rats in IL-4 group than the CON group. The activation of microglia were confirmed through their morphological changes of amoeboid that were more obvious and of a larger number in IL-4 group.

### Neonatal IL-4 Over-Exposure Increased M-CSF Slightly in the Hippocampus on P35 and P49.

To further evaluate hippocampal inflammation induced by neonatal IL-4 over-exposure, another main pro-inflammatory cytokines, including IFN-γ, IL-2, IL-8, IL-17 and M-CSF in the hippocampus, were also detected on P35, P49, P63. These three time points were chosen because they spread the time span from just before the emerging of the hippocampal inflammation, indicated by hippocampal levels of IL-1β, IL-6 and TNF (Fig. 2), and throughout its lasting period.

The data showed that only M-CSF has a slight increase (about 1.1 fold compared with CON mice) on P35 and P49 (Paired samples *t*-test with Bonferroni-Holm correction; P35, *p* < 0.001, Bonferroni–Holm *α* = 0.0167; P49, *p* < 0.001, Bonferroni–Holm *α* = 0.0250; P63, *p* = 0.1220, Bonferroni–Holm *α* = 0.0500; *n* = 15; Fig. 8A). The other cytokines shown no differences between groups (Paired samples *t*-test with Bonferroni-Holm correction, all *p* values > 0.05 and all *p* values > Bonferroni–Holm *α* values; *n* = 15; Fig. 8D–E).

These results suggested that the increased numbers of microglia in the hippocampus on P49 (Fig. 7) may be associated with the higher level of M-CSF in the hippocampus.

## Discussion

This study revealed that neonatal IL-4 over-exposure altered the inflammatory status in the CSF. Specifically, an instant anti-inflammatory cytokine response followed by a subsequent pro-inflammatory cytokine response was induced in the CSF in IL-4 group with more macrophage accumulation in dura mater. Furthermore, there was a positive relationship between the CSF level and the hippocampal level of each of the four cytokines in IL-4 group.

A question arising here is how high level of IL-4 induced delayed inflammation in the CSF and brain parenchyma. Our data supported the conclusion that there was no pro-inflammatory cytokine response in the blood which ruled out the possibility that delayed neuroinflammation was simply transported by the pro-inflammatory cytokines in the blood when the delayed neuroinflammation was on. This suggests that IL-4 could gain access to the CNS and CSF during early life to participate in the inflammation in situ. Thus, we can speculate that the high level of IL-4 can cross BBB and blood-cerebrospinal fluid barrier (BCSFB) into the brain to transmit peripheral inflammatory messages, which then further induce delayed neuroinflammation in the hippocampus, because existing studies have shown that the immune signals (such as cytokines and chemokines) in the brain parenchyma, CSF and blood could be transported among the three different kinds of tissue (Alves de Lima et al. 2020; Erickson and Banks 2011; Wu and Nakanishi 2014; Khaibullin et al. 2017). And our previous study has shown high level of IL-4 can prolong the penetration period of BBB for IL-4 into the hippocampus (Wang et al. 2018). Meanwhile, the disruption of the BCSFB was significantly related to IL-4 in serum, according to Kazim et al. (Ott et al. 2018). Therefore, neonatal over-exposed IL-4 could cross BBB and BCSFB into the brain parenchyma, and is then involved in neuroinflammation. Similar conclusion has been made in the research results by Sun et al., in mice with airway inflammation, circumventricular organ macrophages responding to the high level of IL-4 could release pro-inflammatory cytokines, which then penetrate into the brain through the BBB to promote the production of a second wave of cytokines by microglial cells (Han et al. 2018).

The brain is surrounded by meninges, which house the CSF. The meninges contain a wide repertoire of immune cells, and there is a high proportion of immune cells in the dura (roughly 1 in 10 dural cells are macrophages) (Balcziak and Russo 2022; Rua and McGavern 2018). M1 macrophage is the primary cell type in releasing pro-inflammatory cytokines (such as IL-6, IL-1β and TNF) in the meninges (Balcziak and Russo 2022). We found that neonatal IL-4 over-exposure induced the increase of the levels of pro-inflammatory cytokines, and simultaneously the increased number and activation of macrophages was observed on P42. This result is consistent with previous findings, namely, the cytokines can be transported among the three different tissue as immune signals (Alves de Lima et al. 2020; Erickson and Banks 2011; Wu and Nakanishi 2014; Khaibullin et al. 2017).

However, the interactions of cytokines between brain parenchyma, CSF and blood are bidirectional and sophisticated. Therefore, we have yet to explore the exact mechanism and connection. Nevertheless, a specific phenomenon was observed in this study. Under the IL-4 over-exposure condition, both hippocampus and CSF showed elevated levels of pro-inflammatory cytokines. By comparison, the increase of pro-inflammatory cytokines in CSF is earlier than those in the hippocampus, which suggests that the assessment of inflammatory status in CSF may provide experimental evidence and new clue for developing CSF biomarkers in neuroinflammatory disorders of the brain. In addition, the levels of the three pro-inflammatory cytokines in CSF were positively correlated with those in brain parenchyma on P42, which suggested that determining the levels of pro-inflammatory cytokines could be considered as a potential approach to evaluate the severity of neuroinflammation.

It is possible that the observed effect on the hippocampus is caused by IL-4 entering the brain through the choroid plexus (Han et al. 2018), which has a more permissive barrier to blood components. From there, the cytokine could spread to the brain, modulate/activate the functionality of the glia, and later (P35-P42) lead to the accumulation of the pro-inflammatory cytokines in the cerebrospinal fluid. This prompted us to evaluate the correlation between the serum and hippocampal levels of each cytokine. However, only one cytokine (IL-β) had a significant correlation between its serum and hippocampal levels. This may be because circulating blood flows throughout the body, and thus the levels of cytokines in circulating blood are regulated by more factors than those in the CNS. For example, studies have shown that intestinal dysbiosis or skin injury affects the immune status in circulating blood (Levy et al. 2017; Larouche et al. 2018).

Autism spectrum disorder (ASD) is a neural developmental disability in children that warrants special emphasis in this study (Lukasik et al. 2019). The fact that long-term IL-4 over-exposure events in neonatal period could lead to ASD-like behaviors phenotype was demonstrated in research by Saitoh et al. using an allergic asthma model (Saitoh et al. 2021). Furthermore, epidemiological studies have shown a strong statistical correlation between the risk of ASD and either maternal or infantile allergic/atopic disorders, including asthma, eczema, and food allergies which are often accompanied by elevated IL-4 (Sicherer et al. 2010; Chen et al. 2014; Lambrecht et al. 2019; Patel et al. 2020; Langan et al. 2020). Therefore, IL-4 over-exposure in the early life may be one of the risk factors for ASD.

A growing body of studies has indicated that children with ASD diagnosis have brain pathology suggestive of ongoing neuroinflammation or encephalitis in different regions of brain, indicated by activation of microbial cells and astrocytes, expression of the inflammatory molecules is increased, as well as aberrant expression of nuclear factor kappa-light-chain-enhancer of activated B cells (Hughes et al. 2023). Unfortunately, children with ASD diagnosis are currently not generally assessed for an underlying diagnosis of encephalitis or neuroinflammation. However, it is important to predict and early diagnose encephalitis and neuroinflammation, as the neuroinflammatory disorders are potentially treatable (Kern et al. 2015; Kothur et al. 2016). Our study has shown that the potential neurotoxicity of IL-4 over-exposure in neonatal period and predictive value of evaluating of inflammatory status in CSF on neuroinflammation. Therefore, IL-4 over-exposure should be avoided in the early life. Also, clinicians should strengthen monitoring of the inflammatory state of CSF to detect neuroinflammation as early as possible.

In the early stage of life (from infancy to childhood), the rates of IL-4 over-exposure-related diseases (e.g., asthma, eczema, and aeroallergen sensitization) and food allergy are significantly higher in males (Rosario et al. 2021; Yung et al. 2018; Chen et al. 2003; de Marco et al. 2000; DunnGalvin et al. 2006). These findings could indicate that males are much more likely than females to suffer brain damage induced by IL-4. Therefore, neonatal male rats were used to investigate the effect of elevated peripheral IL-4 on inflammatory condition in the hippocampus and CSF. Specifically, the changes of cytokines in CSF and hippocampus were determined in IL-4 over-exposed rats, as well as the relation between the two kinds of tissue, aiming to evaluate the predictive value of elevated pro-inflammatory cytokines in CSF for neuroinflammation induced by IL-4 over-exposure in the neonatal period.

## Conclusion

In sum, the present study showed that IL-4 over-exposure in the neonatal phase induced delayed neuroinflammation, which suggested that the clinical events resulting into neonatal IL-4 over-exposure may increase risks for neuroinflammation-related diseases. These findings enrich our understanding of the potential neurotoxic effects of neonatal IL-4 over-exposure and suggest a necessity to carry out related clinical studies aiming to develop a possible strategy in the prediction and early diagnosis of pediatric neuroinflammatory diseases related to IL-4 over-exposure.

## Supplementary Information

Below is the link to the electronic supplementary material.

Supplementary file1 (XLSX 32 KB)

## Data Availability

All data generated or analyzed during this study are included in this published article.

## Abbreviations

IL-4: Interleukin-4
CNS: Central nervous systems
BBB: Blood–brain barrier
BCSFB: Blood-cerebrospinal fluid barrier
CSF: Cerebrospinal fluid
rIL-4: Recombinant rat IL-4
PBS: Phosphate-buffered saline
ASD: Autism spectrum disorder
IL-6: Interleukin-6
IL-1β: Interleukin-1β
TNF: Tumor necrosis factor
SSS: Superior sagittal sinus
TS: Transverse sinus
PFA: Paraformaldehyde
